# Using the Healthy Native Youth Implementation Toolbox to Provide Web-Based Adolescent Health Promotion Decision Support to American Indian and Alaska Native Communities: Implementation Study

**DOI:** 10.2196/67885

**Published:** 2025-04-16

**Authors:** Amrita Sidhu, Ross Shegog, Stephanie Craig-Rushing, Nicole Trevino, Michelle Singer, Cornelia Jessen, Gwenda Gorman, Sean Simpson, Melissa Peskin, Belinda Hernandez, Christine Markham

**Affiliations:** 1 Department of Health Promotion and Behavioral Sciences, School of Public Health, UTHealth Houston, 7000 Fannin St, Houston, TX, 77030, United States, 1 8323504322; 2 Northwest Portland Area Indian Health Board, Portland, OR, United States; 3 Alaska Native Tribal Health Consortium, Anchorage, AK, United States; 4 Inter Tribal Council of Arizona, Inc, Phoenix, AZ, United States; 5 Good Medicine Tribal Public Health Consulting, Madison, WI, United States

**Keywords:** implementation, culturally relevant program, evidence-based health promotion, user engagement, reach, decision support system, American Indian, Alaska, native communities, youth, adolescent, decision support, Alaska native, health inequities, sexual, reproductive, mental health, AI/AN, Tribal organization, Google Analytics, toolbox

## Abstract

**Background:**

American Indian and Alaska Native (AI/AN) youth experience numerous health inequities, including those in sexual, reproductive, and mental health. Implementation of culturally relevant, age-appropriate evidence-based programs may mitigate these inequities. However, numerous barriers limit the adoption and implementation of evidence-based adolescent health promotion programs in AI/AN communities.

**Objective:**

This study examines user reach and engagement from 2022 to 2024 of web-based decision support (the Healthy Native Youth [HNY] website and the embedded HNY Implementation Toolbox), designed to increase the implementation of evidence-based adolescent health promotion programming in AI/AN communities.

**Methods:**

Promotional strategies were designed for optimal geographic reach to Tribal organizations, opinion leaders, federal decision makers, and funders. Promotional channels included grassroots, community, and professional networks. We used Google Analytics to examine the uptake of the HNY website and HNY Implementation Toolbox from January 2022 to January 2024. The Toolbox provides culturally relevant tools and templates to help users navigate through 5 phases of program adoption and implementation: Gather, Choose, Prepare, Implement, and Grow. User reach was estimated by demographic characteristics and geographic location; user engagement was estimated by visit frequency and duration, bounce rates, and frequency of page and tool access.

**Results:**

Over the study period, page views of the HNY website and HNY Toolbox increased 10-fold and 27-fold, respectively. Over the 2-year evaluation period since the Toolbox “go live” date, approximately 1 in 8 users of the HNY website visited the Toolbox. The majority of HNY website users were located in Washington (n=1515), California (n=1290), and Oregon (n=1019) and were aged between 18 and 24 (n=1559, 21.7%) and 25‐34 (n=1676, 23.29%) years. Toolbox users were primarily located in California (n=1238), Washington (n=1142), and Oregon (n=986), mostly aged between 35 and 44 years (n=444, 35%). Both website and Toolbox users were primarily female, who accessed the site and Toolbox via desktop computers. The most frequently accessed phase pages within the Implementation Toolbox were Gather, Choose, Implement, and Prepare, as supported by bounce rates and average time on page. The most viewed phase was the “Gather” phase, with 3278 views. The most frequently downloaded tools within the Toolbox were Gather: Community Needs and Resource Assessment, with 136 downloads. The phases and tools accessed may have differed based on the user’s goal or stage of implementation.

**Conclusions:**

Findings indicate positive initial reach and engagement of the HNY website and HNY Implementation Toolbox among AI/AN educators that has consistently increased over the 2 years. The provision of web-based decision support that guides AI/AN users through the adoption, implementation, and maintenance of culturally relevant, age-appropriate, evidence-based adolescent health promotion programs in their communities may help increase the implementation of effective adolescent health promotion programs to ultimately increase health equity among AI/AN youth.

## Introduction

In the United States, as of 2022, the federal government recognizes 574 distinct American Indian and Alaska Native (AI/AN) Tribes across 37 states [1]. Together, the combined AI/AN population comprises 9.7 million people, representing 2.9% of the US population in 2020 [1]. The AI/AN population is young, with 30% being up to 18 years old, compared with 24% of the US total population [2], making adolescent health an important topic in native communities. However, AI/AN youth face many health inequities compared with other racial/ethnic groups, including a disproportionate burden of adverse mental, sexual, and reproductive health, and violence-related outcomes [3-5].

Evidence-based programs (EBPs) that promote adolescent health may help mitigate these inequities among AI/AN teens [6]. An increasing number of EBPs incorporating the strengths and cultural teachings of native communities have been developed or adapted for AI/AN youth [7-13]. Despite efforts to increase access to culturally relevant EBPs, barriers exist that limit their adoption and implementation by AI/AN educators [14-16]. Native communities may lack implementation readiness to discuss and approach adolescent health, experience difficulty navigating Tribal review and school board approval processes [16], have inadequate funding or program integration that compromises program sustainability, or be geographically isolated with limited access to programs [15,17]. Lack of administrative and parental support, lack of trained personnel, and lack of program integration with cultural values represent additional barriers to the implementation of EBPs in this population [15].

**Figure 1.**
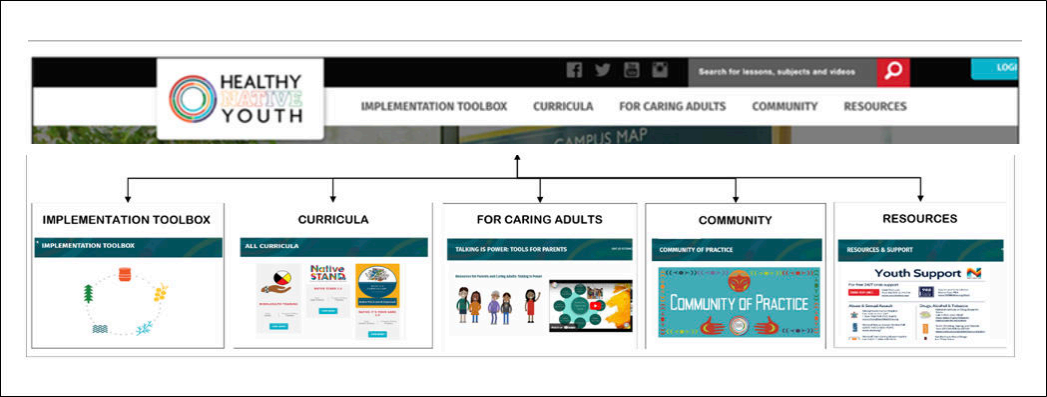
Roadmap of the Healthy Native Youth website landing page (top) and landing pages for each section (bottom) for access by American Indian and Alaska Native (AI/AN) communities.

The Healthy Native Youth (HNY) website was developed in 2016 in collaboration with AI/AN community advisors to mitigate barriers to access and to enhance the adoption and implementation of culturally relevant adolescent health EBPs in native communities to promote health equity [6,14,18,19] (Figure 1). Digital platforms are an accepted delivery channel to enable greater reach of evidence-based interventions to underserved populations at lower costs [20-23]. Digital health interventions for AI/AN youth have been reported to contribute to enhanced health metrics such as mental health, substance use, and sexual risk reduction [10,24]. Prior to the HNY Website, such programs need to be located and accessed individually, without structured dissemination and implementation decision support. The HNY website provides a “one-stop-shop” for Tribal health advocates to access engaging, culturally relevant, age-appropriate health curricula and programs for AI/AN youth within a single website structured to provide implementation decision support [14]. The website comprises 5 sections: “Implementation Toolbox” (guidance for adoption and implementation of EBPs), “Curricula” (information about culturally informed EBPs), “For Caring Adults” (empowerment for nurturing adult-youth communication), “Community” (networking for advocates of adolescent health), and “Resources” (links and materials for youth advocates). The Curricula section of the HNY website contains comparative information on the content, specifications, and access to 19 culturally relevant, age-appropriate evidence-based for AI/AN youth focused on multiple topics, including mental health, sexual health, substance use, and suicide prevention. The user can filter and compare curricula on multiple dimensions to determine “best-fit” for their community and includes implementation materials at no cost.

The HNY Implementation Toolbox, housed within the HNY website is maintained and owned by the Northwest Portland Area Indian Health Board (NPAIHB) and provides stepped web-based decision support designed to facilitate the adoption and implementation of adolescent health EBPs in native communities (Figure 1). The Toolbox was developed on WordPress, an open-source content management system using an iterative design process informed by native practitioners and academicians to adapt an existing web-based decision support system (iCHAMPSS), which is described elsewhere [17,25]. Digital decision support can provide web-based expert guidance to facilitate complex tasks [26]. Findings from a 6-month user experience study with 25 AI/AN educators rated the Toolbox as credible, easy to use, and more helpful than current resources [17]. In the 6-month postsurvey, participants indicated increased knowledge, advocacy skills, and self-efficacy to adopt, implement, and maintain culturally relevant adolescent health promotion programs in their community [17]. The Toolbox launched in January 2022 and the sustainability of the program is through ongoing grant funding through the aforementioned NPAIHB. National Institutes of Health R21 funds (approximating US $440,000 total costs; US $164,000 indirect costs) were sufficient to develop and formatively assess the HNY Website Toolbox.

**Figure 2.**
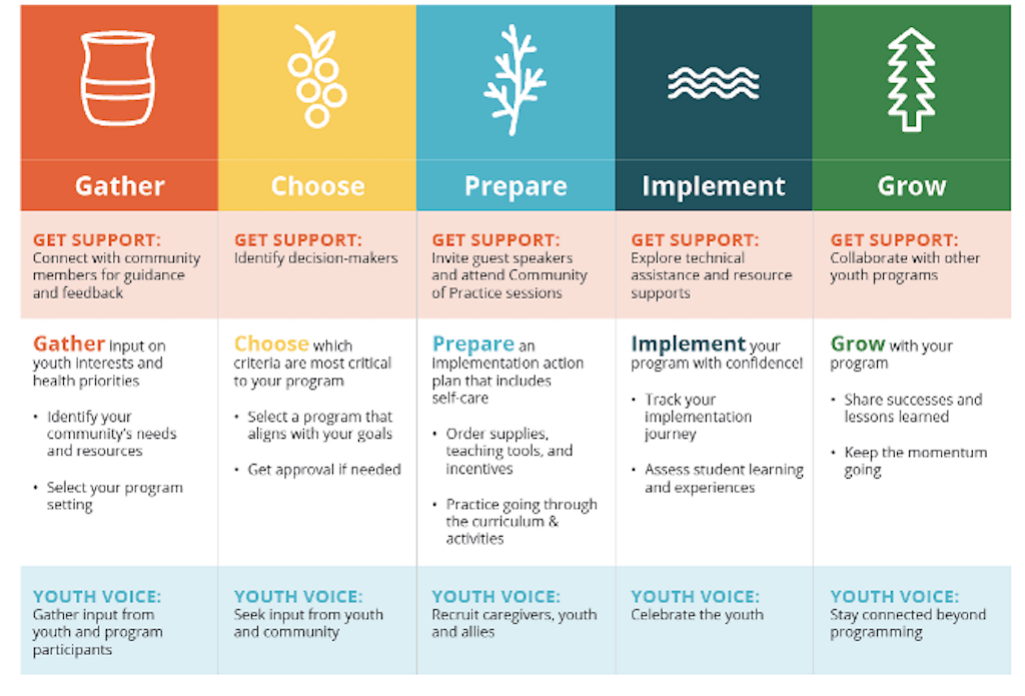
The 5 culturally adapted dissemination and implementation phases and associated tasks in the Healthy Native Youth Implementation Toolbox.

The Toolbox is designed to guide users through five culturally adapted dissemination and implementation phases: (1) Gather, (2) Choose, (3) Prepare, (4) Implement, and (5) Grow. (Figure 2) [17]. The “Gather” phase guides users to identify the health priorities and interests of youth in their community. “Choose” guides users to select a culturally relevant and age-appropriate evidence-based health program for their community. “Prepare” guides users to implement the selected program, including facilitating support from key decision makers. “Implement” guides users to deliver their selected program while emphasizing listening, reflection, and feedback. Finally, “Grow” guides users to sustain their selected program [17]. Each phase includes strategies to generate support for community members, elders, and youth throughout the planning process. The Toolbox comprises a “resource tools library” with over 20 phase-specific support tools, templates, helpful tips, links to web-based resources, and stories from the field (testimonials from experienced native health educators) [17]. All rights, including copyright, for this website and content on this Site are owned by or licensed to the NPAIHB.

The purpose of this study was to examine user reach and engagement with the HNY website and the embedded HNY Implementation Toolbox during an initial 2-year roll-out of the Toolbox from January 2022 to January 2024. Study findings of the reach of these web-based decision support strategies can inform further strategies to increase dissemination and implementation and provide a foundation for future hybrid effectiveness-implementation studies.

## Methods

### HNY Website and Implementation Toolbox Dissemination

Outreach dissemination strategies over the 2-year period encompassed channels through grassroots, community, and professional networks. These information channels comprised monthly AI/AN Community of Practice Network meetings highlighting the HNY Website and Toolkit for professionals who plan, manage, and facilitate healthy youth programs in Indian country, social media posts through existing networks in the AI/AN community devoted to health messages and event promotion, field studies with AI/AN practitioners and community members and related manuscript publication, presentations at professional conferences (eg, American Public Health Association, Department of Health and Human Services Office of Population Affairs Teen Pregnancy Prevention) and Tribal meetings (eg, The Tribal Public Health Conference) reporting formative, outcome, and process data from the HNY Toolbox, and presentations on varied health topics that promoted the HNY website as a resource. Rollouts were planned to ensure optimal breadth (geographically) and depth of exposure (including local Tribal organizations, opinion leaders, national Federal decision makers, and funders).

### Data Extraction and Analysis

Data from the HNY website and the embedded HNY Implementation Toolbox were assessed using Google Analytics collected from January 2022 to January 2024. Google Analytics is a web analytics service that tracks and reports website traffic and user behavior [27,28]. This tool is used extensively by digital marketers and businesses to test and improve digital marketing performance, assess customer behavior, and identify strategies to optimize and improve traffic to a website by providing insight into user behavior.

Data were collected to assess reach, user demographics, and user engagement through a real-time, interactive dashboard accessible through a registered account on the Google Analytics website [29].

Reach is the extent to which an intervention or program is delivered to the priority population and was assessed as the number of users from each US state [29]. It is a necessary antecedent for community exposure to intervention content and any opportunity to establish effectiveness [29,30]. A user was defined as a visitor to the sites who had an assigned unique identifier with one or more associated sessions [29]. User demographics were assessed by geographic location, user gender and age, and the type of device used to access the sites. User engagement was assessed by the number of page views, number of pages viewed per session, number of “tools” downloaded, average duration of sessions, bounce rate, and exit rate. Page views were assessed as the number of separate views each page received. A session was defined as a user visit to, and interaction with, the webpage (eg, button clicks, downloads, and page navigation). Pages per session were assessed as the number of web pages of the site that the user viewed in a single session [29]. Duration of a session was assessed as the average length of time in minutes that users spent on a website/webpage during a session [29]. Bounce rate*,* or nonengagement rate, was assessed as the percentage of single-page sessions a specific webpage received. If a user loaded a page but did not interact a second time (by viewing another page) within 30 minutes, this was considered a bounce [29]. For example, a bounce rate of 50% means that half the users engaged with the website on the visited page only. Exit rates were assessed as the percentage of users that left the website from a webpage (ie, the webpage was the last page visited by them before they exited the website) [29]. For example, an exit rate of 20% means that 20% of users left the website from this page. Reporting bounce rates and exit rates assess if the website/tool met the expectations of a user. If these are lower than industry standards then the site is considered to be engaging [29]. Downloads were assessed by user selections of a download option for a given resource template.

### Ethical Considerations

This study was reviewed and approved by the Committee for the Protection of Human Subjects at the University of Texas Health Science Center at Houston (institutional review board number: HSC-SPH-18‐0958). The HNY website also outlines its privacy policy in the footer section of the website as a means of informed consent from users and describes how user data are collected and may be used for research purposes. All data used are deidentified and anonymous. This manuscript, including related images and supplementary material, does not allow the identification of individual participants or users. No compensation was offered to participants as this paper reports analytics of a web-based resource that is accessible free of cost.

## Results

### HNY Website Analytics

**Figure 3.**
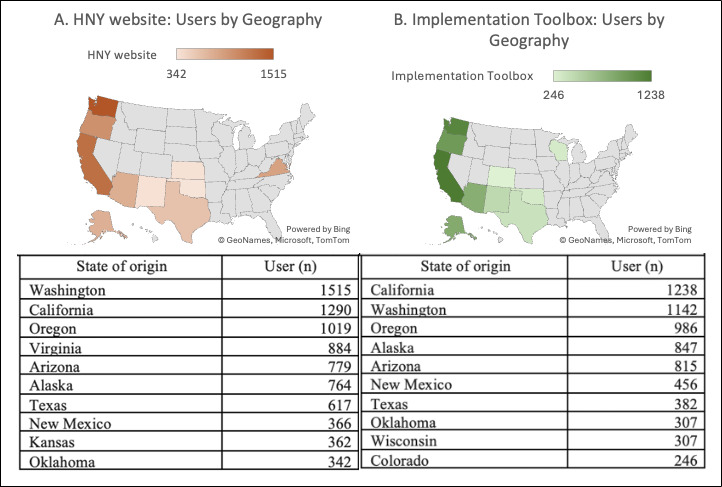
Geographic distribution (top 10 US states) and number of users by state who visited the HNY Website (left column) and HNY Implementation Toolbox (right column) from January 1, 2022, to January 1, 2024.

**Figure 4.**
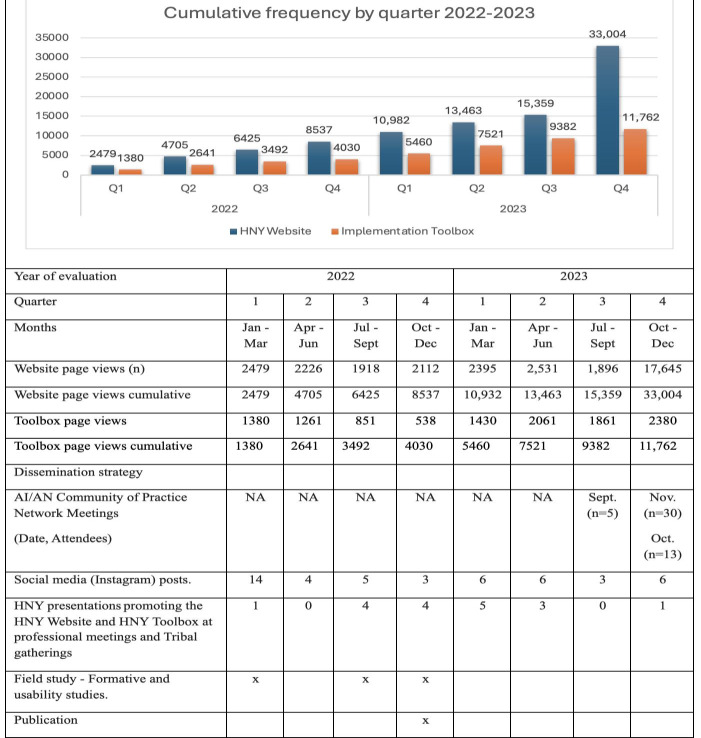
Cumulative user frequency over time by quarter from Jan 2022 to Jan 2024 and associated dissemination strategies (outreach events). AI/AN: American Indian and Alaska Native; HNY: Healthy Native Youth; NA: not available; Q: quarter.

From January 2022 to January 2024 the HNY website had 10,237 total users. Most users were located in Washington (n=1515), California (n=1290), and Oregon (n=1019) (Figure 3A). The majority of users identified as female (n=5217, 70.0%), were mainly 25‐34 years (n=1676, 23.0%) or 18‐24 (n=1559, 22.0%) years of age, and accessed the website primarily via desktop (80.0%) and mobile (19.0%) devices (Table 1). The most frequently accessed sections (in decreasing order) were Curricula (9902 views), Resources (6397 views), HNY Implementation Toolbox home (4364 views), Community (4054 views), and For Caring Adults (3766 views) (Table 2). Page duration ranged from 1.7 minutes (Implementation Toolbox) to 3.06 minutes (Curricula), bounce rate from 50.2% (Curricula) to 65.5% (Community), and exit rates from 15.9% (Curricula) to 60.0% (For Caring Adults). Access to the HNY website increased from 2479 page views in the first quarter of 2022 to a cumulative total of 33,004 page views by the fourth quarter of 2023 (Figure 4).

**Table 1.** User demographics (gender, age, and device used) for the Healthy Native Youth (HNY) website (left column) and HNY Implementation Toolbox (right column) from January 2022 to January 2024.

		HNY website	HNY Implementation Toolbox
Users by gender, n (%)			
Male		2213 (30.0)	244 (34.0)
Female		5217 (70.0)	472 (66.0)
Users by age (years), n (%)			
18‐24		1559 (22.0)	253 (20.0)
25‐34		1676 (23.0)	319 (25.0)
35‐44		1638 (23.0)	444 (35.0)
45‐54		1216 (17.0)	135 (11.0)
55‐64		690 (9.0)	82 (6.0)
65 and older		416 (6.0)	39 (3.0)
Users by device (%)^a^			
Mobile		19.0	19.0
Tablet		1.0	0
Desktop		80.0	81.0

a During the time period of this study, the number of users by device was not a part of the analytic dashboard. Therefore, only percentage values have been reported.

**Table 2.** Most frequently accessed sections in the Healthy Native Youth (HNY) website from January 2022 to January 2024 (Google Analytic data from January 2022 to January 2024).

Section^a^	Total users, n	Page views, n	Page views/session^b^	Page duration^b^ (min)	Bounce rate^c^, %	Exit rate, %
HNY website home	10,237	38,009	2.4	2.75	40.7	19.6
Curricula	3494	9902	3.8	3.06	50.2	15.9
Resources	2849	6397	2.51	2.83	64.6	40.3
Implementation Toolbox home	2566	4364	1.8	1.71	59.8	35.4
Community	2486	4054	1.8	1.3	57.4	60.0
For caring adults	1727	3766	3.64	2.0	65.5	36.6

a Corresponds to top-level sections in the HNY website (Figure 1).

b Average.

c Nonengagement rate.

### HNY Implementation Toolbox Analytics

From January 2022 to January 2024 the Implementation Toolbox had a total of 2566 total users (Table 3) and 11,762 page views (Figure 4). Most users were located in California (n=1238), Washington (n=1142), and Oregon (n=986) (Figure 3B). The majority of users identified as female (n=472, 66%), were mainly 35‐44 years (n=444, 35%) or 25‐34 (n=319, 25%) years of age and accessed the Toolbox mainly via desktop (81%) and mobile (19%) devices (Table 1). Phase page views (in decreasing order) were Gather (3278 views), Choose (1275 views), Prepare (907 views), Implement (843), and Grow (618 views) (Table 3). Page duration ranged from 1.67 minutes (Grow) to 2.08 minutes (Choose), bounce rate from 41.8% (Gather) to 72.9% (Prepare), and exit rates from 22.1% (Prepare) to 34.5% (Grow). The most frequently downloaded tools within the Toolbox were from the Gather phase and included the “Community needs and resource assessment” factsheet (136 views), the “Identify youth advocates and community partners” factsheet (111 views), and a “Bingo data collection” activity guide (83 views) (Table 4). Access to the HNY Implementation Toolbox increased from 1380 page views in the first quarter of 2022 to a cumulative total of 11,762 page views by the fourth quarter of 2023 in conjunction with dissemination activities of scientific presentations, community of practice sessions, social media posts, and webinars (Figure 4).

**Table 3.** Phases of the Healthy Native Youth (HNY) Implementation Toolbox accessed from January 2022 to January 2024 across the United States by user metrics.

Implementation phase	Total users, n	Page views, n	Page views/session^a^	Page duration^a^ (min)	Bounce rate, %	Exit rate, %
Implementation Toolbox landing page	2566	4364	1.8	1.71	60.3	35.5
Gather	1863	3278	16.7	2.07	41.8	33.9
Choose	876	1275	12.3	2.08	49.5	22.3
Prepare	575	907	27.1	1.40	72.9	22.1
Implement	488	843	7.8	1.7	64.0	30.8
Grow	353	618	5.7	1.66	57.8	34.5

a Average.

**Table 4.** Top 10 most frequently downloaded tools from the Healthy Native Youth (HNY) Implementation Toolbox by phase, type, and name from January 2022 to January 2024 across the United States.

#	Implementation phase	Tool type	Tool name	Downloads, n
1	Gather	Factsheet	Community needs and resource assessment	136
2	Gather	Factsheet	Identify youth advocates and community partners	111
3	Gather	Activity sheet	Bingo data collection activity	83
4	Prepare	Template	Curriculum implementation plan template	76
5	Gather	Checklist	Site selection	72
6	Implement	Log	Session reflection log template	44
7	Prepare	Inventory list	Example list of materials to order for native STAND	40
8	Implement	Log	Class attendance sheet template	30
9	Choose	Factsheet	Identify youth advocates and community partners	29
10	Gather	Factsheet	Alaska Tribal Health Consortium adolescent health action plan	28

## Discussion

### Principal Findings

The HNY website and embedded HNY Implementation Toolbox provide web-based resources to support the adoption, implementation, and maintenance of culturally relevant, age-appropriate evidence-based adolescent health promotion programs in AI/AN communities. The HNY website is a “one-stop-shop” for Tribal health advocates to access culturally relevant and age-appropriate health curricula, programs, and related resources for AI/AN youth [14]. The embedded HNY Implementation Toolbox provides guidance to enable them to successfully implement these curricula in their communities. Over the 2-year evaluation period since the Toolbox’s “go live” date, approximately 1 in 8 users of the HNY website visited the Toolbox. This, and expanded geographic reach, corroborate the previously reported utility of the Toolbox [17]. The differential traffic and reach between the HNY website and the Toolbox may reflect the comparative maturity of the HNY website compared with the relatively newer embedded Toolbox. The greater reach in the western states is expected because the HNY website was developed and is housed in the NPAIHB located in Washington State, and early promotion was among affiliated Tribes in Washington State, Oregon, and Idaho. Demographically, the Toolbox users were older, with 35% (n=444) being 35‐44 years of age. This is likely a reflection of the demographics of community health advocates who are evaluating community needs, facilitating decision-making on the curricula best addressing these needs, and actively engaging community support to adopt and implement these curricula in their communities. This, and whether users had attended Tribal health conferences or Community of Practice gatherings in which the Toolbox was promoted could not be verified from the Google data.

Over the evaluation period, page views of the HNY Website and the HNY Toolbox increased 10-fold and 9-fold, respectively. Page views to the Toolbox witnessed a steady increase from 2022 to 2023. The reason for this is uncertain but is possibly attributable to the Toolbox being showcased in monthly Community of Practice meetings that gather dozens of advocates for Tribal youth in Indian country. This suggests the potential for embedded practitioner networks as a particularly utilitarian strategy to disseminate youth programs [27,28]. Given that the HNY website and Implementation Toolbox coexist, the promotion of one likely promotes traffic to the other. This is the strength of a collaborative approach that provides multiple resources that address a particular theme, whether it be a priority population, setting, or health domain. The cumulative page views over time are also suggestive of the need to provide a persistent and longitudinal promotional strategy. We will continue to build awareness nationally via presentations, workshops, and round table discussions at Tribal, education, and public health conferences.

The Toolbox’s average bounce rate (57.7%) was lower than that of the HNY website (62.1%) and also the industry average (62.3%); the average number of Toolbox pages visited per session was almost 5 times than that of the industry average (11.9 vs 2 pages) [14,31,32]. Further, the duration of use on both sites (2.4 and 1.8 respectively) are close to averages on comparable websites (2.58) [33]. These findings suggest that the Toolbox is “sticky,” and that users are finding material commensurate with their needs. This is also supportive of previously reported positive usability ratings for the site from AI/AN community decision makers.

User preferences within the Toolbox were for the Gather Phase and tools related to this phase that enable needs assessment and identification of community advocates. This may be indicative of Tribal communities being in this early phase of adopting and implementing adolescent health programs, that these tools are particularly useful for the work of health educators, or simply that community members are likely to start at the first step in their review and exploration of a novel support tool like the Toolbox. Future studies can investigate if progression through stages occurs over time, the association of phase use with “in-situ” community implementation, as well as challenges to implementation that can be a function of differing state or local policies, regional internet accessibility, funding, community readiness to adopt programs, and logistics of obtaining school board approvals [16].

Study findings need to be interpreted in the context of several methodologic limitations and biases due to data collection. Google Analytics was originally intended for marketing and sales purposes and is limited largely to descriptive data [34,35]. Guidelines for the use and interpretation of Google Analytics do not exist. Discrepancies in the data are possible due to a new client ID being assigned every time a user deletes cookies or signs in from a different device or browser [36]. This may produce an inaccurate count of “new” users because different IDs could be allocated to the same user [34]. Additionally, deletion of cookies may degrade data captured via Google Analytics which relies on user cooperation for successful data collection [20]. Google Analytics assigns a value of 0 seconds if a user visits a webpage until they navigate to a second page, which can lead to inaccurate page duration assessments [34]. Google Analytics does not collect demographic and contextual data on ethnicity, Tribe, or the context of the application of the HNY website and Toolbox. The gender of users captured by Google Analytics may only reflect a subset of users that log into their Google accounts when visiting the page and it is difficult to determine how the demographics align with the target population [34]. Page views and resource downloads do not necessarily equate to the actual adoption and implementation of programs. There is a risk of selection bias wherein the analytics reported may only reflect a subset of users that log into their Google accounts The purpose of use, whether in the context of seeking guidance on program implementation, review and feedback for research, or simply to assuage curiosity, was not ascertained in this study and will need to be if the utility of the HNY Website and Toolbox is to be fully understood. To address the above biases and limitations, reporting using Google Analytics may be applied in combination with other field data collection (qualitative or quantitative) to triangulate data sources. This study reported on an array of promotional strategies to achieve increased dissemination. Future studies, perhaps using a mixed methods design, are recommended to investigate their comparative effectiveness.

Despite limitations, the use of Google Analytics to assess and gather feedback on web-based decision support and to track user behavior on multiple devices is well accepted [20]. Advantages are that it can be used for self-comparison over time and provide a comparison to industry standards. This can provide a positive feedback loop wherein higher website traffic builds positive normative perceptions that generate further user traffic through “word of mouth.” An assessment of reach represents an early phase in understanding program dissemination and implementation that provides the foundation for future hybrid effectiveness-implementation evaluation, including network analysis to assess the dynamics of community diffusion.

### Conclusions

The HNY website and HNY Implementation Toolbox provide AI/AN educators and youth advocates with step-by-step guidance to support the adoption, implementation, and maintenance of culturally relevant, age-appropriate evidence-based adolescent health promotion programs in their communities. Google Analytics provides insight into the use and reach of the HNY website and Implementation Toolbox among AI/AN youth advocates, which will help inform ongoing dissemination efforts. Future studies are needed to assess the Toolbox’s actual impact on the uptake and implementation of adolescent health promotion EBPs in native communities.

## Abbreviations

AI/AN: American Indian and Alaska Native
EBP: evidence-based program
HNY: Healthy Native Youth
NPAIHB: Northwest Portland Area Indian Health Board

## References

[1] Federally recognized American Indian tribes and Alaska Native entities. USA.gov.

[2] (2022). Facts for features: American Indian and Alaska Native heritage month: November 2021. US Census Bureau.

[3] Trends in teen pregnancy and childbearing. Office of Population Affairs.

[4] Allen J, Wexler L, Rasmus S (2022). Protective factors as a unifying framework for strength-based intervention and culturally responsive American Indian and Alaska Native suicide prevention. Prev Sci.

[5] Clayton HB, Kilmer G, DeGue S (2023). Dating violence, sexual violence, and bullying victimization among high school students -youth risk behavior survey, United States, 2021. MMWR Suppl.

[6] Morrison-Beedy D, Mazurek Melnyk B (2019). Making a case for integrating evidence-based sexual risk reduction and mental health interventions for adolescent girls. Issues Ment Health Nurs.

[7] Spencer LM, Schooley MW, Anderson LA (2013). Seeking best practices: a conceptual framework for planning and improving evidence-based practices. Prev Chronic Dis.

[8] Craig Rushing S, Gaston A, Kaufman C, Markham C, Jessen C, Gorman G, Dyson LE, Grant S, Hendricks M (2016). Indigenous People and Mobile Technologies.

[9] Tingey L, Chambers R, Patel H (2021). Prevention of sexually transmitted diseases and pregnancy prevention among Native American youths: a randomized controlled trial, 2016–2018. Am J Public Health.

[10] Shegog R, Craig Rushing S, Jessen C (2017). Native IYG: improving psychosocial protective factors for HIV/STI and teen pregnancy prevention among youth in American Indian/Alaska Native communities. J Appl Res Child.

[11] Hafner SP, Craig Rushing S (2019). Sexual health, STI and HIV risk, and risk perceptions among American Indian and Alaska Native emerging adults. Prev Sci.

[12] Skye M, McCoy T, Kelley A (2021). Effectiveness of Native STAND: a five-year study of a culturally relevant sexual health intervention. Adolescents.

[13] Kaufman CE, Whitesell NR, Keane EM (2014). Effectiveness of circle of life, an HIV-preventive intervention for American Indian middle school youths: a group randomized trial in a Northern Plains tribe. Am J Public Health.

[14] Craig Rushing S, Stephens D, Shegog R (2018). Healthy Native Youth: improving access to effective, culturally-relevant sexual health curricula. Front Public Health.

[15] Sacca L, Shegog R, Hernandez B (2022). Barriers, frameworks, and mitigating strategies influencing the dissemination and implementation of health promotion interventions in indigenous communities: a scoping review. Implement Sci.

[16] Markham C, Torres J, Rushing SC, Gorman G, Jessen C, Gaston A (2018). Usability and psychosocial impact of decision support to increase sexual health education in American Indian and Alaska Native communities. J Health Dispar Res Pract.

[17] Markham CM, Rushing SC, Manthei J (2022). The Healthy Native Youth Implementation Toolbox: using implementation mapping to adapt an online decision support system to promote culturally-relevant sexual health education for American Indian and Alaska Native youth. Front Public Health.

[18] Peskin MF, Hernandez BF, Markham C (2011). Sexual health education from the perspective of school staff: implications for adoption and implementation of effective programs in middle school. J Appl Res Child.

[19] Hernandez BF, Peskin M, Shegog R (2011). Choosing and maintaining programs for sex education in schools: the CHAMPSS model. J Appl Res Child.

[20] Marcu G, Ondersma SJ, Spiller AN, Broderick BM, Kadri R, Buis LR (2022). Barriers and considerations in the design and implementation of digital behavioral interventions: qualitative analysis. J Med Internet Res.

[21] Marcu G, Ondersma SJ, Spiller AN, Broderick BM, Kadri R, Buis LR (2022). The perceived benefits of digital interventions for behavioral health: qualitative interview study. J Med Internet Res.

[22] Linardon J, Cuijpers P, Carlbring P, Messer M, Fuller-Tyszkiewicz M (2019). The efficacy of app-supported smartphone interventions for mental health problems: a meta-analysis of randomized controlled trials. World Psychiatry.

[23] Nelson LA, Spieker AJ, Mayberry LS, McNaughton C, Greevy RA (2021). Estimating the impact of engagement with digital health interventions on patient outcomes in randomized trials. J Am Med Inform Assoc.

[24] Toombs E, Kowatch KR, Dalicandro L, McConkey S, Hopkins C, Mushquash CJ (2021). A systematic review of electronic mental health interventions for Indigenous youth: results and recommendations. J Telemed Telecare.

[25] Peskin MF, Hernandez BF, Gabay EK (2017). Using intervention mapping for program design and production of iCHAMPSS: an online decision support system to increase adoption, implementation, and maintenance of evidence-based sexual health programs. Front Public Health.

[26] Bhargava HK, Power DJ, Sun D (2007). Progress in web-based decision support technologies. Decis Support Syst.

[27] Noar AP, Jeffery HE, Subbiah Ponniah H, Jaffer U (2023). The aims and effectiveness of communities of practice in healthcare: a systematic review. PLoS One.

[28] Bodison SC, Sankaré I, Anaya H (2015). Engaging the community in the dissemination, implementation, and improvement of health-related research. Clin Transl Sci.

[29] Balasubramanian BA, Fernald D, Dickinson LM (2015). REACH of interventions integrating primary care and behavioral health. J Am Board Fam Med.

[30] Reilly KL, Kennedy S, Porter G, Estabrooks P (2020). Comparing, contrasting, and integrating dissemination and implementation outcomes included in the RE-AIM and implementation outcomes frameworks. Front Public Health.

[31] Average bounce rate by industry 2024 [benchmark]. Capturly.

[32] Pages per session: a measure of engagement. Analyzify.

[33] Average session duration: industry benchmarks. First Page Sage.

[34] Analytics tools & solutions for your business. Google Marketing Platform.

[35] Henström M, Duncanson K, Collins CE, Ashton LM, Davidson E, Ball R (2022). Online reach and engagement of a child nutrition peer-education program (PICNIC): insights from social media and web analytics. BMC Public Health.

[36] Client ID vs user ID. Analytics Mania.

